# Poplar Hot Water Extract Enhances Barrier and Antioxidant Properties of Chitosan/Bentonite Composite Film for Packaging Applications

**DOI:** 10.3390/polym11101614

**Published:** 2019-10-04

**Authors:** Mengya Sun, Na Liu, Shuzhen Ni, Huiyang Bian, Yingjuan Fu, Xiaoqian Chen

**Affiliations:** 1 State Key Laboratory of Biobased Material and Green Papermaking, Qilu University of Technology, Shandong Academy of Sciences, Jinan 250353, China; 2 Jiangsu Co-Innovation Center for Efficient Processing and Utilization of Forest Resources, Nanjing Forestry University, Nanjing 210037, China

**Keywords:** chitosan, poplar hot water extract, bentonite, UV blocking ability, antioxidant activity, food packaging material

## Abstract

Herein, the chitosan-based (CS) composite film was fabricated via a simple and efficient blending approach by adding poplar hot water extract (HWE), bentonite (BT) and chitosan. The addition of HWE largely improved the UV blocking ability and antioxidant properties of the resultant composite film, and simultaneously a tortuous path was constructed within the chitosan matrix to enhance the water vapor and oxygen barriers after the addition of BT. Specially, the content of HWE at 10 wt % gave a greatly decreased UV light transmittance at 280 nm to the CS-BT-HWE composite film that was 99.36% lower than that of CS-BT film, and the oxidation resistance was 9.65 times higher than that of CS-BT. The mechanical properties and surface morphological observation evaluated by scanning electron microscopy (SEM) and scanning probe microscope (SPM) confirmed the film had a denser structure. The internal chemical structure analyzed using solid state NMR, FTIR and X-ray spectra exhibited the resultant Maillard structure and strong hydrogen bonding that contributed to the improved mechanical properties. Overall, the as-prepared composite film has great potential as food packaging materials, and also provides a high-efficient utilization pathway for HWE.

## 1. Introduction

Food packaging materials dominated by petroleum-derived polymers pose a huge threat to food safety and the environment. Biodegradable films from renewable sources represent an interesting alternative to conventional plastic materials [1]. Chitosan (CS), as one of the most abundant renewable polymers, has been studied in various fields due to its nontoxicity, biodegradability, biocompatibility, and excellent film-forming ability [2]. These unique characteristics have increased the interest of using chitosan as a raw material to develop biodegradable films intended for food packaging materials. However, pristine chitosan films possess a generally weak mechanical barrier, and antioxidant properties, which greatly limit their practical application [3,4]. Therefore, researches have been focused on the development of methodologies to improve the properties of these biopolymer-based materials [5].

As an essential necessity to improve the corresponding properties of chitosan regarding its use in food packaging, the incorporation of micro or nanofillers into the chitosan matrix [6] or blending with other biopolymers have been utilized efficiently as practical methods [7,8]. Bentonite (BT) is mainly composed of montmorillonite (about 85%~90%, Na_x_(H_2_O)_4_{(Al_2_~_x_Mg_x_)[Si_4_O_10_](OH)_2_}), which comprises two sheets of tetrahedral silica layers with one alumina octahedral layer in the middle, and is the most commonly used natural inorganic mineral in fabricating functional materials [9]. It can be uniformLy dispersed in a chitosan matrix to form a chitosan/bentonite composite. Even at low clay additions (a few wt %), the entire composite can exhibit as a compact structure, with the majority of polymer chains residing in close contact with the clay surface [10]. Therefore, it can significantly improve the mechanical strength, water vapor and gas barrier properties of the chitosan film [11,12,13].

Hemicellulose is an amorphous branched heteropolysaccharide with various compositions in different raw material types. Xylan is the main component of hemicelluloses in hardwoods and grass-type biomass (like wheat straw or rice straw), while the main components of hemicellulose in softwoods is galactomannans [14]. Xylan extracted from corn cobs was co-heated with chitosan to prepare a polysaccharide-based food preservative in Li’s study [15]. They also found that the resultant product showed good scavenging ability and reducing ability, as well as improved antibacterial property [16]. Purified hemicellulose exhibits excellent performance in the preparation of composite materials. However, from an implementation and commercialization perspective, it is critical to produce functional products with less refined resources and mixed reagents rather than highly purified biopolymers [17].

Hot water pretreatment has been regarded as an environmental-friendly pretreatment process for improving cellulose digestibility of lignocellulosic materials, while it produces hot water extract (HWE) as a by-product [18]. The HWE from hardwood chips contains a large amount of xylan with a low degree of polymerization (in the range of generally 2~25) and xylose, a certain amount of low molecular weight lignin and lignin–carbohydrate complex [19,20]. Due to the presence of a large amount of hemicellulose-derived sugars in the HWE, they can be fermented to xylitol and ethanol. However, the fermentation inhibitors in HWE, such as furfural, acetic acid and lignin derivatives, impede the efficiency of the fermentation process [21,22]. Commonly, the hemicellulose-derived sugars and lignin can be efficiently utilized only after concentrating the hydrolysis liquor due to their dilute nature, which poses another difficulty for the presence of the filter blockage and fouling. Therefore, it is still a challenge to convert HWE into value-added products through a cheap and simple approach [23]. In fact, few studies have directly used HWE for preparing functional materials. Chen et al. first attempted to introduce BT and the reinforcing agent carboxymethyl cellulose (CMC) into the hot water wood extraction matrix to produce HWE/BT/CMC nanocomposite film, which exhibited good heat-resistance, excellent gas barrier properties, and high mechanical strength [7]. Then they combined HWE with BT as a basic component with low addition of graphene oxide to produce a ternary bio-excited nanocomposite film with high strength and fire resistance properties [24].

In this work, a simple approach is provided to utilize the renewable forest product waste (hot water wood extract) via blending with the CS-BT composite film to prepare CS-BT-HWE bio-nanocomposites with excellent oxidation resistance and barrier properties. The novelty of this work is to eliminate the time-consuming process of purifying HWE, and the HWE can be directly used to improve the performance of chitosan composite film. The barrier properties (UV light blocking capacity, water vapor and oxygen barrier properties), mechanical strength, water solubility and antioxidant activity were evaluated accordingly. The as-prepared film could be suitable for food packaging and barrier materials.

## 2. Materials and Methods

### 2.1. Materials

CS with 90.07% degree of deacetylation and 1.8226 × 10^5^ of viscosity-average molecular weight was purchased from Sinopharm Chemical Reagent Co., Ltd. (Shanghai, China). BT was obtained from Thermo Fisher Scientific Co., Ltd. (Fairlawn, OH, USA). Five-year-old poplar wood chips of *Populus × euramericana ‘Neva’* were obtained from Shandong Huatai Paper Co., Ltd. (Shandong, China). The size of the chips was selected as 2.0–3.5 cm long, 1.5–2.0 cm wide, and about 0.5 cm thick.

### 2.2. Preparation of Hot Water Wood Extract (HWE)

The pretreatment step was carried out in a horizontal cooking pot (Kumagai Riki Kogyo Co., Ltd., Kumagai, Japan) with 30 g of poplar chips (absolute dryness) and a liquid–solid ratio (L: S) of 6:1. The temperature was raised to 180 °C at a heating rate of 2.02 °C·min^−1^. The hot water containing the wood extract and wood chips was separated after preheating for 90 min. The pre-hydrolysis factor for the pretreatment of poplar chips by hot water extraction used in this paper was 2079, which was calculated according to the formula in the literature [25]. Subsequently, the hot water wood extract was obtained after centrifuging at 5000 rpm for 10 min and removing the insoluble impurities. The as-prepared HWE was stored at 4 °C in the refrigerator. The resultant HWE was obtained from the poplar chips through one-time hot water extraction. The component analysis of HWE was conducted. Results showed that the hemicellulose-derived sugars in the HWE had an average molecular weight (*M*_w_) of about 258 g/mol, and consisted of oligosaccharide, monosaccharide (mainly xylose) and ~13% (wt) lignin. Information on the composition of the HWE has been provided in Tables S1 and S2.

### 2.3. CS-BT-HWE Composite Film Preparation

The composite films were prepared by the casting method. First, 1% (*w*/*v*) CS solution was prepared by dissolving CS in 1% aqueous acetic acid solution at room temperature with stirring of 350 rpm for 8 h. Subsequently, BT suspension was prepared using the procedure modified from the literature [12]. Firstly, 2% BT (wt) suspension was achieved under magnetic stirring for 30 min, then it was centrifuged by an AXTG16 centrifuge (Jiangsu, China) at 100 rpm for 5 min to remove the sediment. After purification, the exfoliation was proceeded by a SCIENTZ-IID ultrasonic dispersion instrument (Zhejiang, China) at 500 W for 30 min. Further, the suspension was centrifuged again (3800 rpm, 10 min) to remove the insoluble clay aggregates. The TEM image and particle size distribution diagram of BT showed that the BT is nano-sized (Figure S1a,b). Afterwards, BT was added dropwise to a chitosan solution (1%, *w*/*v*) at a ratio of 5% (*w*/*w*) under continuous magnetic stirring, followed by adding different loading of HWE with 10%, 20%, 30% and 40% (*w*/*w*) to the mixture. The reaction was carried out for 10 h with stirring at room temperature, and then poured into a polystyrene petri dish (diameter of 90 mm) and dried at 45 °C for 10 h. Before characterization, the films were kept at 25 °C and a relative humidity (RH) of 67% for 48 h to balance the moisture content. The pure chitosan and the HWE–chitosan solutions were prepared following the same procedures except the steps involving BT were skipped. The mass of the casted film was between 0.3~0.345 g.

### 2.4. Color Properties

The surface color of the film was measured using a chroma meter (Xrite i1 PRO Spectrophotometer, Granville, MI, USA) with a white color plate (L* = 97.75, a* = −0.49 and b* = 1.96) as a standard background. Hunter color values (L*, a* and b*) were ascertained by taking an average of five readings from each film sample. The total color difference (∆*E*) was calculated as follows:

(1) $$∆E={\left[{\left(∆L^{*}\right)}^{2}+{\left(∆a^{*}\right)}^{2}+{\left(∆b^{*}\right)}^{2}\right]}^{0.5}$$

where ∆L*, ∆a* and ∆b* are the variations between the color parameters of the film samples and the color standard plate was used as a film background.

### 2.5. Optical Property

Optical property of the composite films was determined by UV–Vis spectra. A rectangular piece of each film sample (5 cm × 5 cm) was directly mounted between the two spectrophotometer magnetic cell holders. The transmittance spectra of the films were measured at selected wavelength ranges from 200 to 800 nm using a UV–Vis–NIR spectrophotometer (Cary 5000 UV–Vis–NIR, American Varian Technology Co., Ltd., Palo Alto, CA, USA). The optical properties of pure chitosan and composite films were characterized by the transmittance of UV (280 nm) and visible (660 nm) regions. Three measurements were made for each sample and the average values were recorded.

### 2.6. Antioxidant Activity

The antioxidant activity of the film was evaluated using DPPH (2, 2-diphenyl-1-picrylhydrazyl) free radical scavenging assay according to Blois with slight modification [26]. A total of 0.5 g of the membrane was placed in 100 mL of deionized water and stirred at 40 °C for 1 h, then 1 mL of the supernatant was thoroughly mixed with 3 mL of 0.1 mM DPPH solution (of which the solvent was the methanol). The solution was incubated in the dark for 30 minutes and then the absorbance was measured at 517 nm. The percentage of DPPH free radical quenching activity was determined using the following equation:

(2) $$\mathrm{DPPH}\;\mathrm{radical}\;\mathrm{scavenging}\;\mathrm{effect}\;\left(\%\right)=\frac{A_{0}-A_{i}}{A_{0}}\times 100$$

where *A*_0_ is the absorbance value at 517 nm of the methanolic solution of DPPH and *A*_i_ is the absorbance value at 517 nm for the sample extracts. Each sample was assayed at least three times.

### 2.7. Water Vapor Permeability (WVP) and Oxygen Permeability (OP)

The water vapor permeability of the films was estimated gravimetrically according to the standard method of ASTM E96-95 with a slight modification [27]. In this method, the test film was covered on the Petri dish filled with distilled water. The water mass lost from the dish was monitored as a function of time, and the water vapor transmission rate (WVTR) was calculated from the steady-state region by using a water vapor permeability tester (W3/060, Labthink Co., Ltd., Jinan, China). The measurements were carried out at 38 °C with 90% relative humidity. The water vapor pressure at the outer side of the film was 44.73 mm-Hg. A molecular sieve was used as an adsorbent inside the dish to adsorb all water molecules passing through the film and to maintain partial water vapor pressure. The WVP coefficient of the films was calculated by following equation:

(3) $$\mathrm{WVP}=\frac{WVTR\times d}{∆P}$$

where *d* is the average film thickness (mm); Δ*P* is the partial water vapor pressure gradient.

OP of all film samples was measured by a gas permeability tester (VAC-V2, Labthink Co., Ltd., Jinan, China) according to the testing standard GB/T1038-2000. The sample was placed in the testing holder having an exposed testing area of 28.27 cm^2^ and the average value was taken. The results of the gas permeability test are expressed as a coefficient of permeability (cm^3^·cm/cm^2^·s·Pa). All the analyses were carried out in triplicate.

### 2.8. Water Solubility (WS) and Free Amino Group

Water solubility of the films was determined according to the method described by Souza et al. [28]. Film specimens from each treatment were cut into a rectangle (2 cm × 2 cm) and dried at 45 °C for 24 ± 1 h. The WS of the film was calculated according to the following equation:

(4) $$\mathrm{WS}\left(\%\right)=\frac{W_{1}-W_{2}}{W_{1}}\times 100$$

where *W*_1_ is weight of film after drying at 45 °C and cooling in a desiccator; *W*_2_ is the weight of dried film after immersing in water for 24 ± 1 h.

The content of free amino group in the film was determined by colloid titration method [29]. Each film (0.20 g) was dissolved in 99.80 g of a 5 wt % acetic acid solution at room temperature for 3 h, and the insoluble solid was removed by centrifugation. Then, 1 g of the supernatant was mixed with 30 mL distilled water. The filtrate was titrated with N/400 sodium polyvinylsulfate. The content of the free amino group in the solution was obtained, and relative amount of free amino groups in the film was calculated based on the value of pure chitosan film. All the analyses were carried out in triplicate.

### 2.9. Mechanical Properties

According to ISO 527-3:1995, IDT(GB/T1040.3-2006), the composite film was cut into a rectangle of 10 mm × 80 mm, and the tensile strength and elongation at break were measured on an electronic universal material testing machine (Instron 5963, Boston, MA, USA). The initial clamping distance of the test was 50 mm, and the test speed was 50 mm/min. The measurements were conducted based on fifteen replicates for each sample.

Thickness of the composite films was measured by a manual micrometer (SYNTEK, Jiangsu, China). At least six measurements were performed to different locations for each sample and the average values were calculated and regarded as film thickness.

### 2.10. Scanning Electron Microscopy (SEM) and Scanning Probe Microscope (SPM)

The cross-sectional of the film sample was investigated by using scanning electron microscopy (Regulus8220, Hitachi Co., Ltd., Tokyo, Japan) at 20 kV of accelerating voltage. Micrographs of the surface of the films were obtained by using a scanning probe microscope (Multimode8, Bruker Co., Ltd., Madison, WI, USA), with fixed resolution (256 × 256 data points).

### 2.11. XRD

The composite film was tested using an X-ray diffractometer model (D8-ADVANCE, Bruker Co., Ltd., Bremen, Germany) equipped with Ni radiation (40 kV, 30 mA), 2θ angle range of 5°–45°, scan rate at 2° min^−1^ and sampling interval of 0.02°.

### 2.12. FT-IR

Fourier-transform infrared (FT-IR) spectrometry was carried out to observe the structural interactions of chitosan films incorporated with BT and HWE. The sample and KBr were thoroughly ground and compressed at 1:100 (*w*/*w*) and tested using a Fourier-transform infrared spectrometer (VERTEX70, Bruker Co., Ltd., Karlsruhe, Germany), and the corresponding spectra were recorded in the range from 4000 to 600 cm^−1^ at 4 cm^−1^ resolution and 32 scans per sample.

### 2.13. ^13^C-NMR

Solid-state ^13^C-cross-polarization (CP)/magic-angle spinning (MAS) NMR spectra were recorded on an Agilent 600 DD2 spectrometer (American Agilent Technologies Co., Ltd., Palo Alto, CA, USA) at resonance frequency of 150.72 MHz for ^13^C using the cross-polarization (CP), magic-angle spinning (MAS), and a high-power ^1^H decoupling. The rotor rotational speed was 10 kHz, and the 90° pulse width was 4.2 *μs*. The chemical shift of ^13^C of tetramethylsilane (TMS) at 0 ppm was used as the external reference, and all experiments were carried out at room temperature at 293 K.

### 2.14. Statistical Analysis

A statistical analysis of data was performed through a one-way analysis of variance using Statistical Analysis System (SPSS), version 22, and differences among mean values were processed by the Tukey test. Significance was defined at *p* < 0.05.

## 3. Results

### 3.1. Physical Appearance and Color

**Figure 1:**
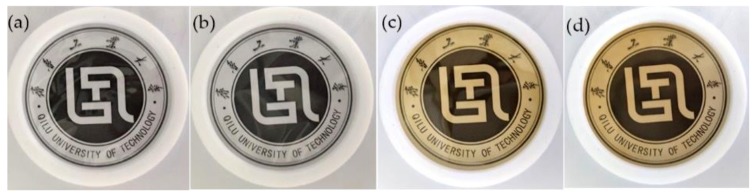
Photograph of (**a**) chitosan (CS), (**b**) CS-bentonite (CS-BT), (**c**) CS- hot water extract (HWE_10_), and (**d**) CS-BT-HWE_10_.

All of the prepared films were visually uniform, free of cracks, transparent, and had a smooth surface, as shown in Figure 1. Due to the uniform dispersion of the nano-sized BT in the chitosan matrix, the transparency of the nanocomposite film with low BT concentration was well preserved and the color was unchanged. By contrast, the HWE incorporated transparency of the film decreased slightly, with a yellowish hue. The effects of HWE concentration on film color and light transmittance properties are shown in Table 1. Adding HWE into CS-BT composite films significantly affected (*p* < 0.05) L* (lightness/darkness), a* (redness/greenness) and b* (yellowness/blueness) values of the film surface. Films without HWE were lighter (higher L* value). When the HWE content increased from 0% to 40%, L* values of the films decreased from 84.03 ± 0.31 to 46.42 ± 1.68, a* increased from 0.92 ± 0.08 to 27.75 ± 1.64 (indicator of the tendency towards redness) and b* values increased from –2.14 ± 0.37 to 63.12 ± 2.06 (indicator of the tendency towards yellowness). It was shown that the HWE addition of 10% lowered the ΔE value significantly (*p* < 0.05), and it was consistent with the observation in the composite film’s color which turned out to be a reddish brown (Figure 1). In addition, the obviously enhanced browning phenomenon was seen after 10 h of reaction at room temperature as compared with the initial CS-BT-HWE mixture solution before reaction (Figure S2). The reason may be that the Maillard reaction forms colored compounds such as brown nitrogenous polymers [29,30]. Moreover, Kanmani [31] reported that lower brightness (L*) values may help prevent oxidative deterioration in packaged foods.

### 3.2. UV Light Barrier Property

**Figure 2:**
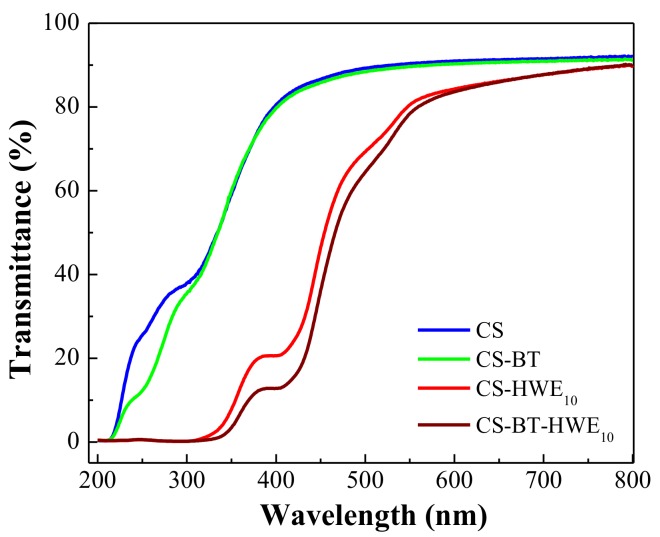
UV–Vis transmittance spectra for pure chitosan films, CS-BT, CS-HWE_10_ and CS-BT-HWE_10_ composite films.

The optical properties of pure chitosan and its composite films were determined by measuring the transmittance in 200–800 nm, and the results are shown in Figure 2. In the ultraviolet light (UV light) wavelength region of less than 310 nm, the transmittance of the CS-BT film was slightly lower than that of the pure CS film. It may be that BT acts as an inorganic particle to reflect and scatter UV light, thereby reducing the UV light transmittance. The addition of HWE into chitosan films significantly decreased (*p* < 0.05) UV light transmittance of the films. Meanwhile, the absorbance of the corresponding solution for the film formation at 280 nm before and after the reaction was recorded, as shown in Figure S3. The reason may be the inherent UV-absorbing groups in the lignin structure, such as phenolic units, ketones and other chromophores [32,33]. In addition, the intermediate compound of the Maillard reaction (hydroxymethylfurfural, furfural) also could contribute to the intensity of peaks at 280 nm [34,35]. In our work, the effect of the HWE addition on the transmittance of the composite film in the UV (280 nm) and visible (660 nm) light regions was recorded. As shown in Table 1, when the amount of HWE added was 10%, the decreased UV light transmittance value at 280 nm of the CS-BT-HWE_10_ film reached 99.36% (calculated from the formula of (T_CS_-_BT_ -T_CS-BT-HWE_)/T_CS-BT_). The greatly decreased transmittance at 280 nm of HWE-added films in the UV light range can confer these films excellent barriers to prevent the lipid oxidation induced by UV light when they are applied in food packaging systems [36].

The transmittance of the CS-BT film without HWE in the visible light region (660 nm) was 90.70 ± 0.16%. The incorporation of 10% HWE into the CS-BT film gave a slightly decreased light transmittance of 86.16 ± 0.31%. It is worth noting that as the content of HWE increased, the transparency of the film decreased, consistent with the literature results [37]. As the concentration of HWE increased, there was no significant difference in the UV light transmittance of the film (*p* ≥ 0.05). In order not to excessively sacrifice the transparency of the film, and to maintain the lower UV light transmittance of the film, it was optimal to add 10%.

### 3.3. Antioxidant Activity 

**Figure 3:**
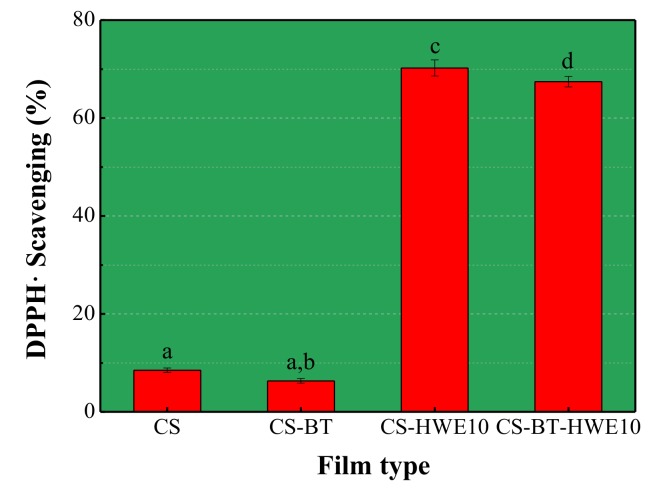
DPPH scavenging ability of the pure chitosan and composite films (*n* = 3). Values are given as mean ± standard deviation. Different letters indicate significantly different (*p* < 0.05) when analyzed by the Tukey test.

DPPH radical scavenging ability was used to indicate the antioxidant activity of the film. This assay is based on the ability of DPPH radical, a stable free radical, to be quenched and thereby decolorize in the presence of antioxidants resulting in a reduction in absorbance values, and the extent of the decoloration depends on the hydrogen-donating ability of the antioxidants [26,38]. As shown in Figure 3, the DPPH radical scavenging activity of the films containing HWE was significantly improved (*p* < 0.05). Among them, the radical scavenging capacity of the CS-BT-HWE_10_ composite film was 9.65 times higher than that of the CS-BT film. It is well known that HWE contains various sugars and lignin degradation products. The possible reasons are as follows: (1) The Maillard reaction between the amino group of chitosan and the carbonyl group of a reducing sugar (xylose), and the produced reducing ketone in the reaction can destroy the radical chain by providing a hydrogen atom, thereby exhibiting excellent DPPH radical removal capacity [15,29]. (2) The total phenolic compounds produced by the degradation of lignin in the extract also have DPPH radical scavenging ability [39]. The pure chitosan films showed scavenging activity on DPPH radical to some extent. It could be attributed to the fact that the free radical can react with the residual free amino (NH_2_) groups to form stable macromolecule radicals, and the NH_2_ groups can form ammonium (NH_3_^+^) groups by absorbing a hydrogen ion from the solution [40].

### 3.4. Water Vapor and Oxygen Permeability

Water vapor and oxygen are the two main penetrants studied in food packaging applications because they can be transferred from the internal or external environment through the packaging film, leading to oxidative deterioration of the food [41]. Good moisture and oxygen barriers in food systems can improve the product quality and shelf life.

Compared with the film without HWE (pure chitosan film and CS-BT film), the addition of BT resulted in a significant decrease (*p* < 0.05) in vapor permeability values (Table 2), which is in good agreement with the current literature [2,6]. Since the silicate layers are completely and homogeneously dispersed in the polymer matrix, they make a tortuous path which acts as an obstruction against water vapor or oxygen transmission [2]. After HWE was incorporated into the film matrix, WVP and OP values did not change largely between the films with or without BT (*p* ≥ 0.05). Thus, BT is the main reason for the correspondingly improved barrier properties of CS-BT-HWE_10_ film. The slightly decreased WVP and OP values in the HWE-incorporated film (*p* ≥ 0.05) (Table 2), compared with the pure chitosan film, might be attributed to the hydrogen bonding and covalent bonding between the chitosan network and HWE (hemicellulose-derived sugars; lignin), that makes the resulting film become dense [42].

### 3.5. Water Solubility Property and Free Amino Group

Degradability of materials after contact with aqueous food substrates is characterized by measuring the solubility of the membrane in water [43]. Without the incorporation of HWE (CS-BT), the BT reduced (*p* < 0.05) the water solubility of the bio-nanocomposites (Table 2), consistent with the results of the literature report [44]. When BT was incorporated, the solubility in water changed little between the films with or without BT (*p* ≥ 0.05), but significantly lower than the CS-BT, indicating that HWE is the main cause of the significant decrease in the water solubility of CS-BT-HWE_10_. It may be that there is a strong interaction between the polysaccharide or lignin from the HWE and the chitosan polysaccharide chain, which reduces the accessibility of the amino and hydroxyl hydrophilic groups in the polymer, thereby enhancing the tolerance of the composite films in water [29].

The change in the relative amount of free amino groups in each film was observed by colloidal titration, and the results are shown in Table 2. The addition of BT decreased the content of the free amino group rapidly, due to the reason that CS showed electropositive with –NH_3_^+^ in the acid solution [45], thus the BT which carried –OH on the edge could form hydrogen bonds (–OH·· ^+^NH_3_–) and gave a decrease to the free amino group content [12]. The addition of HWE reduced the free amino group content significantly (*p* < 0.05), confirming the reduction progress between the reducing sugar and chitosan in HWE, which consumed a large amount of amino groups [46]. At the same time, the relative value of the free amino group in CS-BT-HWE_10_ is the lowest, indicating that both BT and HWE can effectively bind to the amino group of chitosan, which is also one reason for the excellent physical properties of CS-BT-HWE_10_.

### 3.6. Mechanical Properties

A food packaging film is required to maintain its integrity in order to withstand the stress that occurs during shipping, handling and storage [47]. The tensile strength (TS) and elongation at break (EB) values of all films were shown in Table 2. For the neat chitosan film, the tensile strength and the elongation percentage were 39.32 MPa and 11.74%, respectively. The addition of BT resulted in a pronounced enhancement of strength (*p* < 0.05) and a dramatic decrease in the elongation at break (*p* < 0.05) of all CS-BT systems. The increase in the TS of such nanocomposite films can be attributed to the high rigidity and aspect ratio of the nanoclay as well as the high affinity between the biopolymer and the clay, while the decrease in EB can be attributed to the fact that BT restricted the mobility of CS macromolecular chains [48]. As the HWE entered the chitosan matrix, the tensile strength increased from 39.32 ± 2.22 to 49.07 ± 3.46 MPa, and the elongation at break decreased from 11.74 ± 1.70 to 5.14 ± 1.09. The improvement in the tensile strength of the films incorporated with HWE may be attributed to the interaction between the chitosan matrix and native lignin or lignin–carbohydrate complexes in the HWE [7].

Further, when the BT and HWE were incorporated together into the chitosan film matrix, the tensile strength of the composite film reached 59.07 MPa (Table 2). Compared with the addition of BT or HWE to the CS film matrix, the composite material obtained by blending the three components has better mechanical properties. The interaction between HWE or BT and the amino group of chitosan, as well as the strong intermolecular hydrogen bond between HWE and BT [7], act together to contribute to the significantly higher mechanical properties of the film than the other three (*p* < 0.05).

### 3.7. Microstructure

**Figure 4:**
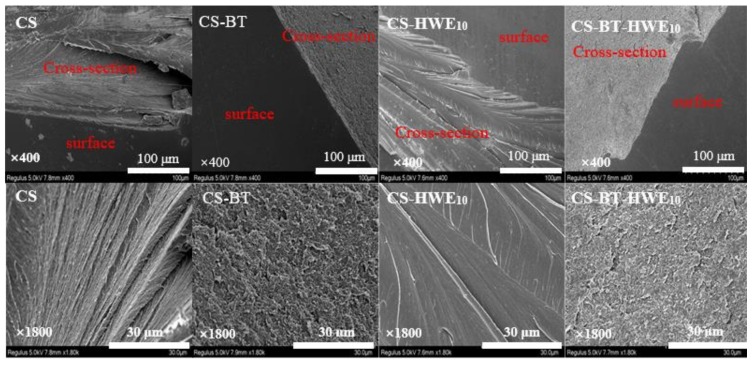
SEM images of composite films prepared from chitosan, BT, and hot water wood extract. The pictures in the first line show the surface and cross-sectional morphology of the films, and the magnified cross-sectional morphology for the corresponding films are in the second line.

The SEM cross-sectional images for the pure chitosan and the nanocomposite films are depicted in Figure 4. In the cross section of the film containing BT, the layered structure can be clearly observed, and it is evident that the BT nanoclay is well dispersed in the matrix. TEM images showed that most of the BT sheets were uniformLy and tightly distributed in the horizontal direction, while a small number of BT sheets existed vertically (Figure S1c). The addition of BT contributed to the improved barrier performance for the composite film due to the increased torturous path. Further, the addition of HWE made the layer structure of the ternary film more compact and presented excellent interfacial adhesion. This may be the reason why the composite films CS-BT-HWE_10_ exhibited much higher tensile strength, which was confirmed in the results of mechanical properties.

**Figure 5:**
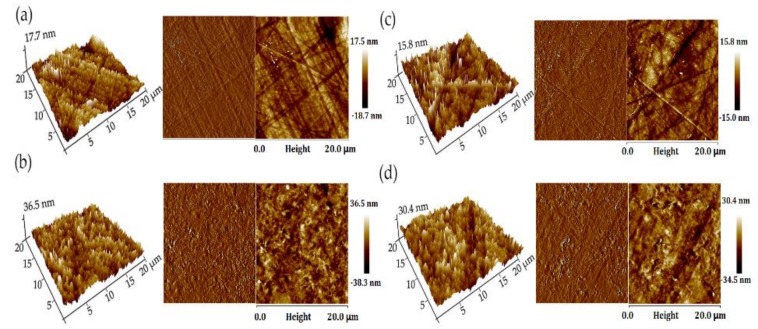
Scanning probe microscope (SPM) images of (**a**) CS, (**b**) CS-BT, (**c**) CS-HWE_10_ and (**d**) CS-BT-HWE_10_. The scanning scale is 20 × 20 μm.

The SPM topography images of chitosan and chitosan nanocomposite films are shown in Figure 5. Through the SPM image, it can be seen that the fiber-like molecule is intertwined on the surface of the pure chitosan film. The addition of HWE did not make obvious changes in the surface characteristics of the CS composite film without the addition of BT. When the BT was incorporated, the original network structure on the surface of the chitosan film was not observed. The reason may be that it was covered by the flaky layer structure of montmorillonite, and the surface roughness significantly increased (*p* < 0.05) [49]. This is the reason why the barrier properties of the composite films are remarkable after the addition of BT.

### 3.8. XRD

**Figure 6:**
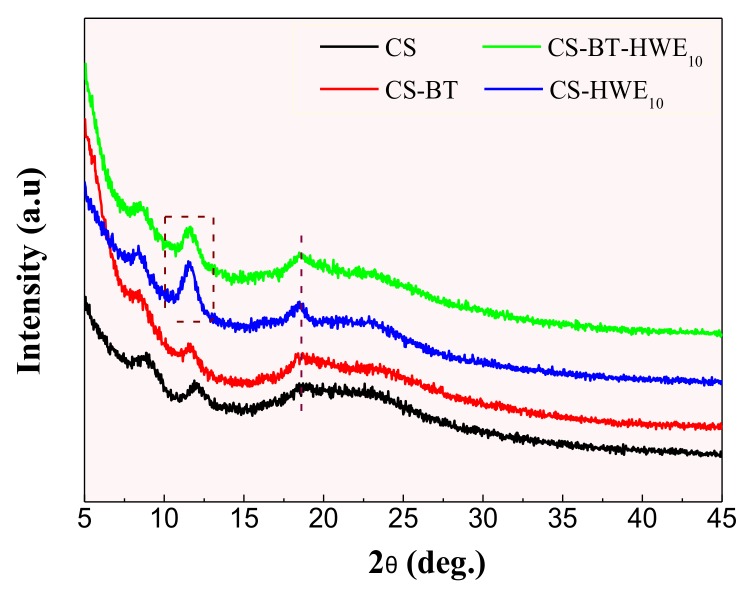
X-ray diffraction patterns of composite films prepared from chitosan, BT, and hot water wood extract.

The XRD patterns of pure CS and its nanocomposites are illustrated in Figure 6. Pure chitosan film showed characteristic peaks around 2θ = 8.87˚, 12.16˚, and 18.92˚. The first two peaks represent the hydrated and anhydrous crystalline structure, respectively, while the latter peak indicates the amorphous structure of chitosan [50,51,52]. It was noticed that incorporation of BT did not significantly affect the crystalline structure of chitosan, as supported by the observation that the XRD diffraction pattern of the CS-BT composites still kept the characteristic peaks of pure chitosan, but the intensity of the diffraction peak decreased slightly. This is because the interaction between CS and BT leads to a decrease in the intermolecular interaction between the blended chains. 

It is worth noting that the incorporation of HWE into the membrane matrix (CS or CS-BT) causes the diffraction peaks of chitosan to move at a low angle and is significantly enhanced. Among them, the diffraction peak increased significantly at 18.49°, indicating that HWE is well miscible with chitosan or bentonite at the molecular level [53]. The improved crystallinity of the films may be the reason that the composite films CS-BT-HWE_10_ exhibited much higher tensile strength, which was confirmed in the section concerning the tensile testing. At the same time, the literature indicates that the anhydrous crystalline peak of chitosan at 11.62° is related to the water resistance of the membrane material [54]. The stronger the intensity of the anhydrous crystalline peak, the stronger the water resistance of the film and the poorer the water solubility, which is consistent with the experimental results of water solubility.

### 3.9. FTIR

**Figure 7:**
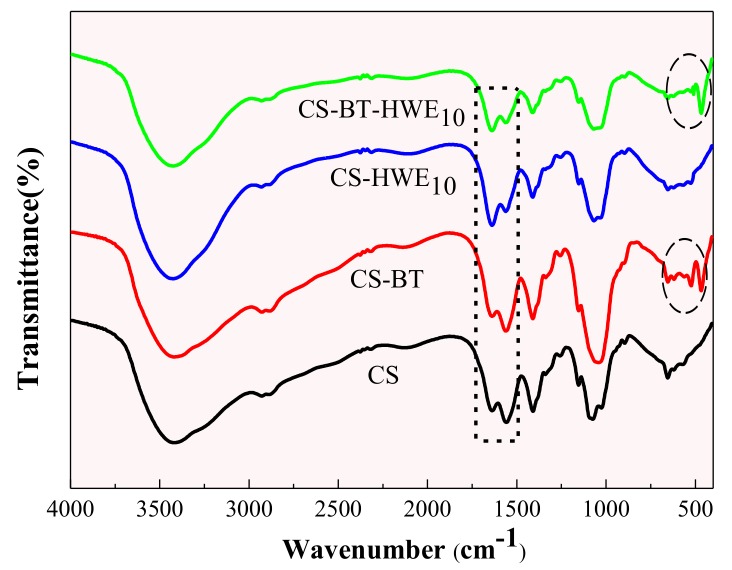
FT-IR spectra of CS, CS-BT, CS-HWE_10_ and CS-BT-HWE_10_ composite film.

Figure 7 shows the FT-IR spectra of CS and the composite films. The adsorption bands of chitosan around 1637 cm^−1^ and 1558 cm^−1^ are attributed to amide I (C = O) and free amino group (–NH_2_), respectively. In the CS and CS-BT composites, the intensity of the peak for the amine band is stronger than the peak of the carbonyl band [55]. Compared to the material without BT, CS-BT and CS-BT-HWE_10_ are characterized by two new absorption bands at 526 cm^−1^ and 469 cm^−1^, corresponding to the coupled vibration of Si–O–M (M is a cationic metal in BT) and M–O of BT [56], respectively. After the addition of HWE to the chitosan matrix, the intensity of the peak for the carbonyl band became stronger than that of the amine band. The same situation was also observed in CS-BT-HWE_10_. These observations demonstrate the interaction between HWE and chitosan, but the interaction preferably occurs at the amine site [57], that is, the Maillard reaction consumes a large amount of amino groups [58]. At the same time, it is consistent with the measurement results of the content of the free amino group.

### 3.10. ^13^C-NMR

**Figure 8:**
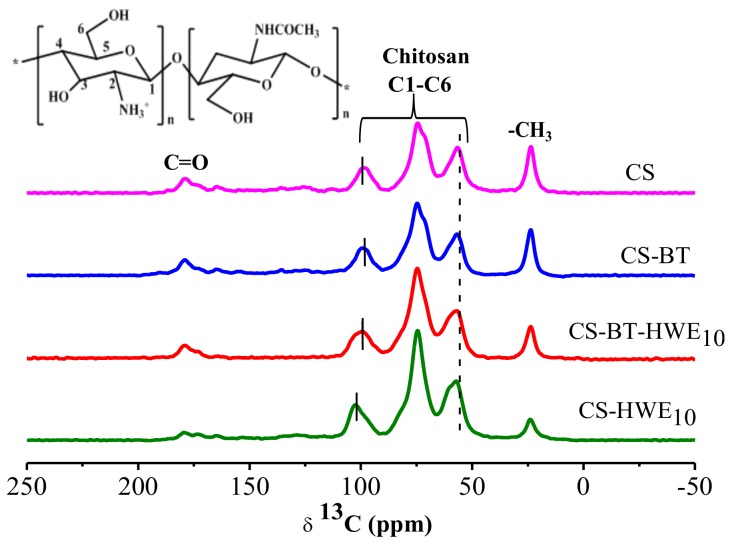
^13^C-NMR spectra of composite films prepared from chitosan, BT, and hot water wood extract.

Chitosan and chitosan composite films were investigated by ^13^C solid state nuclear magnetic resonance in order to better understand the interactions that occur between the bio-polymers at the molecular scale. The corresponding spectra of the composite films are displayed in Figure 8. Firstly, some chemical shifts can unambiguously be assigned to the chitosan polymer revealing the resonance of the cycle (C1–C6), the carbonyl (C = O) and the methyl (CH_3_) [59].

As shown in Figure 8, the addition of BT or HWE changes the chemical shift in the carbon spectrum, that is, the chemical environment around the carbon atom of the chitosan changes. Among them, in the process of chitosan film formation, the acetic acid molecule first destroys the β-1,4 glycosidic bond in the chitosan molecule to produce a positive carbon ion, and then the negatively charged BT or HWE reacts with the positive carbon ion, thus the electronegativity around C1 and C4 increases its displacement to the lower field [60]. Simultaneously, chitosan forms intramolecular and intermolecular hydrogen bonds in the blend system, and the deshielding effect of the hydrogen bonds cause the C2 and C4 resonance positions to shift to the low field. In CS-HWE, the chemical shift of C2 changed from 56.596 to 57.223 ppm. It may be that carbonyl-ammonia condensation reaction between chitosan and hemicellulose-derived sugars in HWE leads to an increased electronegativity around C2 and further shift to the lower field [15,29]. Compared with CS-HWE_10_, the chemical shift of C2 in CS-BT-HWE_10_ shifts slightly to the low frequency, it might be caused by the reaction of BT with the amino group that may influence the progress of the Maillard reaction.

## 4. Conclusions

This study demonstrated that poplar hot water extract (HWE) has great potential for application as high value-added additives in food packaging materials. XRD and SEM results showed that HWE, BT and CS can be well blended to obtain a uniform, thin, yellow and transparent film. Solid-state NMR confirmed the Maillard reaction between xylose from the poplar hot water extract and chitosan. The HWE self-component provided excellent antioxidant activities and UV blocking ability to the films. When 10% of HWE was added, the UV light transmittance at 280 nm of the CS-BT-HWE_10_ film is 99.36% lower than that of CS-BT film, and the oxidation resistance is 9.65 times higher than that of CS-BT. Further, the incorporation of HWE with BT together remarkably improved the tensile properties of the film, and also obviously decreased the water solubility. Although the HWE incorporation did not significantly modify the water vapor and oxygen barrier property of the films, this disadvantage was offset by the addition of BT. Results show that HWE and BT play their respective advantages and contribute to the improved performance of the composite film. Thus, this study demonstrates that the addition of natural extracts and inorganic reinforcement (e.g., BT) to bioactive biopolymers has great potential for being developed into functional packaging material for food and is a promising substitute for synthetic materials.

## Figures and Tables

**Table 1:** Summary of the color and optical properties of composite films with different concentrations of HWE (BT/CS = 5%).

HWE/CS (*w*/*w*)	L*	a*	b*	Δ*E*	*T*_280_	*T*_660_
0%	84.03 ± 0.31 ^a^	0.92 ± 0.08 ^a^	–2.14 ± 0.37 ^a^	7.58 ± 0.31 ^a^	26.37 ± 1.52 ^a^	90.70 ± 0.16 ^a^
10%	66.12 ± 1.82 ^b^	3.69 ± 1.63 ^b^	51.93 ± 2.96 ^b^	54.57 ± 3.56 ^b^	0.17 ± 0.01 ^b^	86.16 ± 0.31 ^b^
20%	57.90 ± 1.66 ^c^	17.69 ± 1.86 ^c^	69.22 ± 0.49 ^c^	75.77 ± 0.99 ^c^	0.09 ± 0.00 ^bc^	78.89 ± 0.35 ^c^
30%	52.22 ± 3.41 ^d^	25.59 ± 2.56 ^d^	64.77 ± 1.18 ^d^	76.96 ± 1.30 ^c,d^	0.09 ± 0.00 ^b,c,d^	70.52 ± 0.38 ^d^
40%	46.42 ± 1.68 ^e^	27.75 ± 1.64 ^e^	63.12 ± 2.06 ^d,e^	79.35 ± 0.97 ^e^	0.09 ± 0.00 ^b,c,d,e^	68.52 ± 0.66 ^e^

Each value is the mean of three replicates with the standard deviation. Superscript letters (**a**–**e**): Within each parameter, values in the same line not sharing lowercase superscript letters indicate statistically significant differences among formulations (*p* < 0.05).

**Table 2:** Barrier properties, water solubility properties and mechanical properties of composite films.

Sample Code	WVP × 10^−13^ (g·cm·cm^−2·^s^−1^·Pa^−1^)	OP × 10^−7^ (cm^3^·cm·cm^−2^· s^−1^·Pa^−1^)	WS (%)	Relative Amount of Free Amino Groups (%)	TS (MPa)	EB (%)	Thickness (μm)
**CS**	8.71 ± 0.20 ^a^	0.95 ± 0.05 ^a^	17.84 ± 0.76 ^a^	--	39.32 ± 2.22 ^a^	11.74 ± 1.70 ^a^	45.14 ± 1.35 ^a^
**CS-BT**	6.72 ± 0.40 ^b^	0.46 ± 0.02 ^b^	15.24 ± 0.33 ^b^	93.26 ± 0.32 ^b^	52.96 ± 4.73 ^b^	7.95 ± 1.23 ^b^	46.43 ± 1.72 ^a,b^
**CS-HWE_10_**	7.91 ± 0.37 ^c^	0.92 ± 0.02 ^c^	11.44 ± 0.38 ^c^	32.28 ± 0.61 ^c^	49.07 ± 3.46 ^b,c^	5.14 ± 1.09 ^c^	48.86 ± 1.07 ^c^
**CS-BT-HWE_10_**	7.13 ± 0.08 ^b,d^	0.68 ± 0.01 ^d^	10.03 ± 0.15 ^c,d^	26.05 ± 1.38 ^d^	59.07 ± 5.22 ^d^	5.51 ± 1.04 ^b,d^	51.71 ± 1.11 ^d^

Superscript letters (**a**–**d**): within each parameter, values in the same line not sharing lowercase superscript letters indicate statistically significant differences among formulations (*p* < 0.05). WVP: water vapor permeability; OP: oxygen permeability; WS: water solubility; TS: tensile strength; EB: elongation at break.

## Supplementary Materials

The following are available online at https://www.mdpi.com/2073-4360/11/10/1614/s1, Table S1: Concentration of hemicellulose-derived saccharides in poplar hot water extract. (HWE concentration is 22.6 g/L), Table S2: Compositions (wt % total saccharides) of poplar hot water extract, Figure S1: The TEM image of BT (a), particle size distribution diagram of BT (b), CS-BT_10_ (c), CS-BT-HWE_10_ (d), Figure S2: The presentation for the color of the mixture solution with different amounts of HWE added in the CS-BT-HWE system. (a) the water-BT-HWE system of which the chitosan in the CS-BT-HWE system was substituted by an equal volume of deionized water; (b) the initial CS-BT-HWE mixture solution before reaction; (c) the CS-BT-HWE system reacted at room temperature for 10 h (From left to right, the HWE amount is 10%; 20%; 30%, 40%, respectively.), Figure S3: The absorbance of the initial CS-BT-HWE mixed solution () and mixing for 10 h () (n = 3).
