# Supplementary material for: Poplar Hot Water Extract Enhances Barrier and Antioxidant Properties of Chitosan/Bentonite Composite Film for Packaging Applications
Source: Polymers (Basel). 2019 Oct 4;11(10):1614. doi: 10.3390/polym11101614 (PMC6836026; doi:10.3390/polym11101614)
Supplement: Supplementary file 1 [file polymers-11-01614-s001.pdf]

Poplar Hot Water Extract Enhances Barrier and Antioxidant Properties of Chitosan/Bentonite Composite Film for Packaging Applications

Mengya Sun [1], Na Liu [1,*], Shuzhen Ni [1,2], Huiyang Bian [2], Yingjuan Fu [1] and Xiaoqian Chen [1]

[1] State Key Laboratory of Biobased Material and Green Papermaking, Qilu University of Technology, Shandong Academy of Sciences, Jinan 250353, China; sunmengyade@163.com (M.S.)

[2] Jiangsu Co-Innovation Center for Efficient Processing and Utilization of Forest Resources, Nanjing Forestry University, Nanjing, 210037, China; hybian1992@njfu.edu.cn (H.B.)

* Correspondence: liuna@qlu.edu.cn (N.L.)

Table S1 Concentration of hemicellulose-derived saccharides in poplar hot water extract. (HWE concentration is 22.6 g/L)

	Concentration (g/L)		Concentration (g/L)
xylose	6.77	Oligo-xylose	3.75
glucose	0.75	Oligo-glucose	1.29
galactose	0.66	Oligo-galactose	0.32
mannose	0.41	Oligo-mannose	0.20
arabinose	0.19	Oligo-arabinose	--

Table S2 Compositions (wt % total saccharides) of poplar hot water extract.

	Compositions (wt %total saccharides)		Compositions (wt %total saccharides)
xylose	47.23	Oligo-xylose	26.18
glucose	5.20	Oligo-glucose	9.02
galactose	4.59	Oligo-galactose	1.40
mannose	2.88	Oligo-mannose	2.21
arabinose	1.29	Oligo-arabinose	--

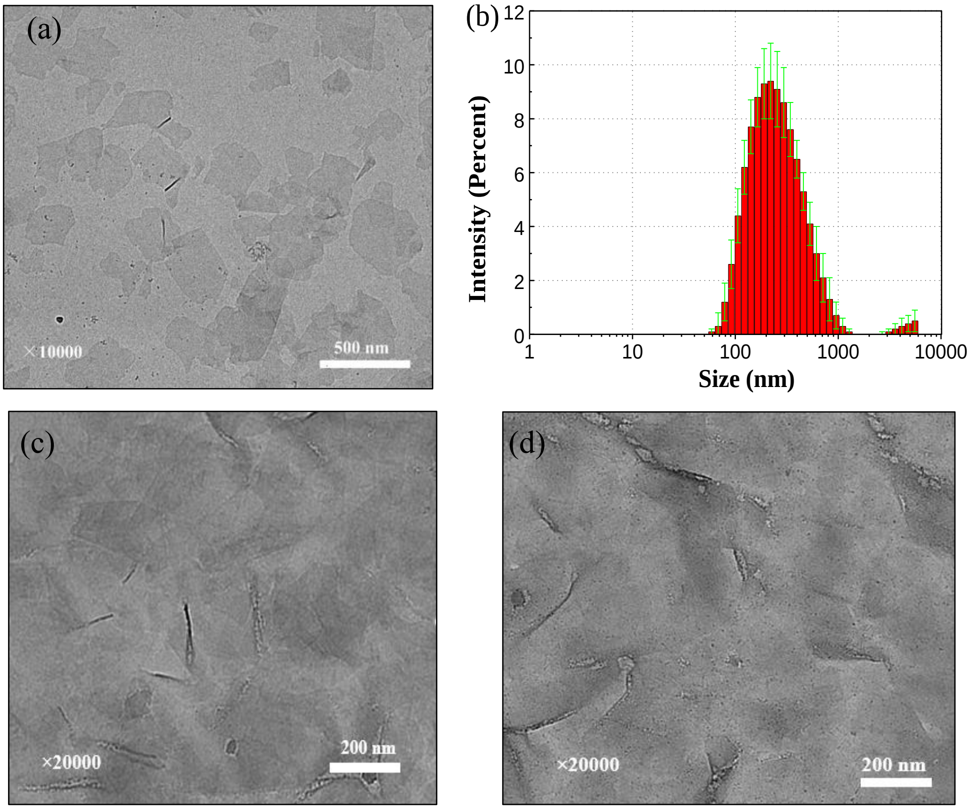

Figure S1. The TEM image of BT (a), particle size distribution diagram of BT (b), CS-BT_{10} (c), CS-BT-HWE_{10} (d).

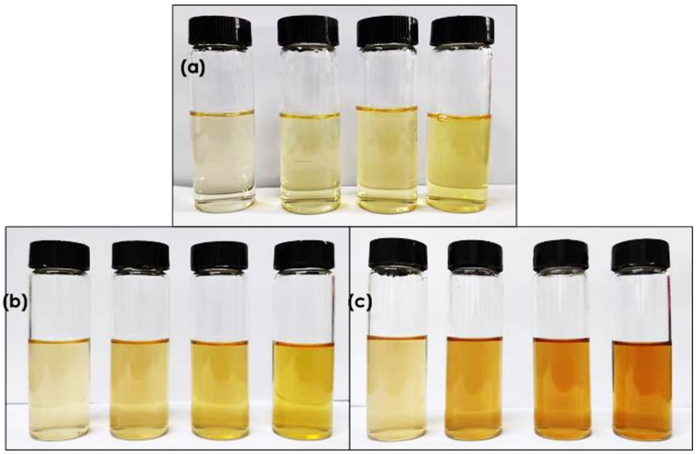

Figure S2. The presentation for the color of the mixture solution with different amounts of HWE added in the CS-BT-HWE system. (a) the water-BT-HWE system of which the chitosan in the CS-BT-HWE system was substituted by an equal volume of deionized water; (b) the initial CS-BT-HWE mixture solution before reaction; (c) the CS-BT-HWE system reacted at room temperature for 10 h (From left to right, the HWE amount is 10%; 20%; 30%, 40%, respectively.)

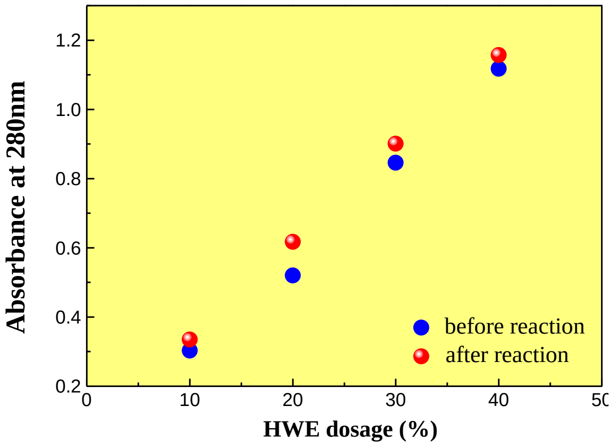

Figure S3. The absorbance of the initial CS-BT-HWE mixed solution (●) and mixing for 10 h (●). (n=3)
